# Bridging the Gap Between the ED and Home: The Community Paramedic‐Led Transitions Intervention for Persons Living With Dementia

**DOI:** 10.1111/jgs.70403

**Published:** 2026-04-15

**Authors:** Meghan Jenkins Morales, Stephanie Ricketts, Corita R. Grudzen, Abraham A. Brody, Joshua Chodosh, Keith Goldfeld, Manish N. Shah, ED‐LEAD Investigators, Lauren Abbate, Rebecca Anthopolos, Alicia Arbaje, Fernanda Bellolio, Jonathan Berkowitz, Andrea Blome, Erik Jonathan Blutinger, Justin Kenneth Brooten, Reed Caldwell, Christopher Caspers, Nathaniel Chin, Edward Cisek, Valerie Cotter, Jeremy Thomas Cushman, Dan David, Allison L. Ducharme‐Smith, Scott Dresden, Mary Ellen Dupont, Marie Carmelle Elie, Andra Farcas, Bucky Ferozan, Jori Fleisher, Nicholas Genes, Cameron Gettel, Andrea Gilmore‐Bykovski, Scott Goldberg, Elizabeth Goldberg, Satheesh Gunaga, Heather Heaton, Courtney Jones, Maura Kennedy, Timmy Li, Joseph Miller, Brian Mittman, Kei Ouchi, Brian W. Patterson, Jennifer Portz, Alex Quinones, Ashley Shreves, Michelle Simpson, Silas Smith, Payal Sud, Joe Suyama, Scotty Thomas, Victoria Vaughan Dickson, Ian Wittman, Nancy Wood

**Affiliations:** ^1^ BerbeeWalsh Department of Emergency Medicine University of Wisconsin School of Medicine and Public Health Madison Wisconsin USA; ^2^ Division of Supportive and Acute Care Services Memorial Sloan Kettering Cancer Center New York New York USA; ^3^ Division of Geriatric Medicine and Palliative Care New York University Grossman School of Medicine New York New York USA; ^4^ NYU Rory Meyers College of Nursing New York New York USA; ^5^ VA New York Harbor Healthcare System New York New York USA; ^6^ Department of Population Health New York University Grossman School of Medicine New York New York USA

**Keywords:** care transitions, community paramedicine, ED‐to‐home transition, emergency department, persons living with dementia, post‐ED outcomes

## Abstract

More than 6 million persons living with dementia (PLWD) in the United States rely on the emergency department (ED) for unscheduled care, with up to half discharged home after treatment. The ED‐to‐home transition poses significant challenges for PLWD and their care partners (referred to as “dyads”), contributing to high rates of ED revisits and adverse outcomes. The Community Paramedic‐led Transitions Intervention (CPTI) was developed to address these challenges by adapting the validated Care Transitions Intervention for the ED setting. Delivered by trained community paramedics, CPTI is a short‐term 30‐day program that includes one home visit and up to three follow‐up phone calls. Using a coaching model, paramedic coaches work with members of the dyad to strengthen their knowledge, skills, and confidence to manage their health and successfully navigate the health care system. CPTI is being implemented as part of Emergency Departments LEading the Transformation of Alzheimer's and Dementia Care (ED‐LEAD), a cluster‐randomized pragmatic trial testing 3 interventions designed to improve outcomes for PLWD discharged home from the ED across 14 health systems and 79 EDs nationwide. This paper describes the CPTI model as implemented within ED‐LEAD, detailing its theoretical foundation, structure, training curriculum, workflow integration, and implementation monitoring. This framework can provide a model for health systems, provider groups, and emergency medical service agencies interested in adopting this innovative approach and implementing the CPTI. Insights from its implementation within ED‐LEAD will guide future efforts to improve post‐ED outcomes and continuity of care for PLWD and their care partners.

## Summary

- Key points
  ○ Persons living with dementia (PLWD) frequently use the emergency department (ED) for unscheduled care and experience significant challenges during the ED‐to‐home transition.
  ○ The Community Paramedic‐led Transitions Intervention (CPTI) is a structured, coaching‐based program delivered by community paramedics that includes a home visit and follow‐up calls to support PLWD and care partners during the 30 days after ED discharge.
  ○ This paper details a rigorously developed and scalable implementation framework for delivering the CPTI across diverse ED settings.
- Why does this matter?
  ○ Improving the ED‐to‐home transition for PLWD is a national priority, yet few evidence‐based models exist to support PLWD and care partners after ED discharge. By describing how the CPTI has been implemented as part of the Emergency Departments LEading the Transformation of Alzheimer's and Dementia Care (ED‐LEAD) study, this paper provides a scalable model for those interested in adopting this innovative approach.

## 1 Background and Significance

The 6 million persons living with dementia (PLWD) in the United States heavily rely on the emergency department (ED) for acute, unscheduled illness care, with 34%–55% using the ED at least once annually [1, 2, 3]. After treatment, approximately half of these patients are discharged home, whether they live in an independent dwelling, senior living community, or other settings [2, 3]. During the ED‐to‐home transition, patients and their care partners, herein referred to as “dyads,” must quickly understand how to care for themselves [4, 5, 6, 7], including managing medication changes; navigating the outpatient care system to obtain follow‐up; understanding and following through on self‐care instructions; and comprehending “red flags,” symptoms for which they should seek further medical attention [8]. Dyads may face greater struggles if care partners lack sufficient knowledge, resources, training, and/or access to in‐home supportive care to effectively manage the many complex care needs of PLWD with an acute illness [9, 10]. These challenges contribute to high rates of ED revisits among PLWD within 30 days (22%–58%) [2, 11, 12], with only 33.8% obtaining necessary outpatient follow‐up care within 7 days and 62.8% within 30 days [2, 11, 12, 13, 14].

As the population of PLWD grows to a projected 13.8 million by 2050, national leaders have identified improving the ED‐to‐home transition as one of the four most important issues for PLWD receiving ED care [15]. Unfortunately, a scoping review found that of the seven studies focused on improving the ED‐to‐home transition for PLWD, only one demonstrated a meaningful improvement in outcomes [16]. That study, performed by our group, involved a pre‐planned subgroup analysis of older adults with impaired cognition (*N* = 81) from a randomized controlled trial testing a transitions intervention for older adults discharged from the ED [4]. This intervention, which we call the Community Paramedic‐led Transitions Intervention (CPTI), is based on the Care Transitions Intervention, a validated and widely implemented hospital‐to‐home transitions program that consists of a hospital visit, a home visit, and three telephone visits to address four pillars of transitional care: (1) medication management; (2) outpatient follow‐up; (3) red flags; and (4) a personal health record [17]. Notably, unlike many medical interventions, the Care Transitions Intervention and CPTI take a coaching approach to support patients.

We found that receipt of the CPTI reduced the odds of ED revisits within 30 days by 75% among patients with impaired cognition [4]. We also found that among all older adults, CPTI increased the odds of in‐person follow‐up with outpatient clinicians within 1 week of ED discharge and increased recollection of red flag symptoms [18]. This prior work provides an important foundation to decrease avoidable healthcare use and morbidity among PLWD discharged from the ED. With these early signs of benefit, we must develop rigorous evidence to support effective, feasible, and dementia‐specific transitional care models that can be implemented in real‐world ED settings.

The CPTI, further adapted to support the ED‐to‐home transition for PLWD, is now being tested in a cluster‐randomized, multifactorial pragmatic trial called Emergency Departments LEading Transformation of Alzheimer's and Dementia Care (ED‐LEAD) [19]. This paper describes the CPTI for PLWD, including the comprehensive training curriculum and associated workflow modifications, to assist health systems interested in adopting this innovative new model of care to better support PLWD being discharged from the ED (For more information on ED‐LEAD, see the related editorial in this issue by Grudzen et al.).

In this paper, we first outline the CPTI conceptual model and its theoretical underpinnings, followed by an analysis of the specific roles of the Program Coordinator and Community Paramedic coaches. We then describe the training curriculum and integrated ED‐to‐home workflow, concluding with the implementation monitoring framework designed to ensure model fidelity and sustainability.

## 2 Intervention Design

### 2.1 CPTI in ED‐LEAD: Overview

When adapting the Care Transitions Intervention to develop the CPTI for ED‐LEAD, we made the following specific modifications: (1) replaced the hospital visit with a brief introduction to the CPTI at ED discharge, delivered by the provider and/or nurse; (2) integrated an enrollment phone call after ED discharge to schedule the home visit; (3) employed community paramedics as coaches; and (4) trained paramedics to specifically coach the dyad and work with PLWD [4].

### 2.2 CPTI in ED‐LEAD: Conceptual Model

**FIGURE 1:**
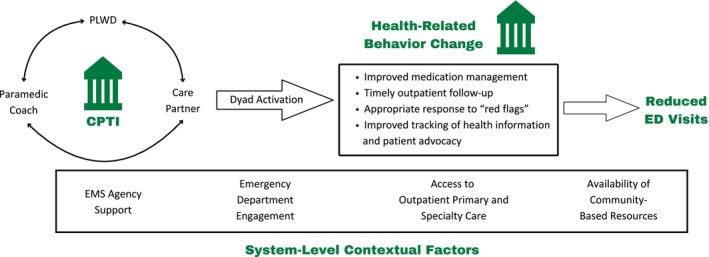
CPTI conceptual model. CPTI, Community Paramedic‐led Transitions Intervention; ED, emergency department; EMS, emergency medical services; PLWD, persons living with dementia.

The CPTI conceptual model (Figure 1) is guided by the Theory of Planned Behavior [20, 21] and aims to promote health‐related behavior changes that reduce ED revisits. The intervention is grounded in the triadic encounter, in which the PLWD, care partner, and paramedic coach collaborate toward shared goals. The CPTI content is organized around the same four pillars discussed earlier [17]. During each encounter, the coach engages the PLWD and care partner as a team, using motivational interviewing techniques to build their confidence and motivation to manage their health effectively.

The purpose of the triadic encounter is to “activate” the dyad. To do this, the coach engages both the PLWD and care partner in actively managing the health and healthcare of the PLWD. Activation is critical because evidence suggests that patients and care partners who are more knowledgeable, confident, and engaged are more likely to adhere to recommended care plans, recognize and respond to health issues promptly, and navigate the healthcare system effectively [22]. In the context of CPTI, activation occurs through goal setting, skill building, and collaborative problem‐solving with the coach. In alignment with the four pillars, dyad activation leads to improved medication management, timely follow‐up with outpatient care, appropriate recognition and response to “red flags,” enhanced tracking of health information (personal health record), and patient advocacy within the healthcare system. These behavior changes are expected to reduce avoidable ED use. The CPTI conceptual model also recognizes that system‐level contextual factors influence CPTI effectiveness. For example, if outpatient care is inaccessible, timely follow‐up may not be feasible, limiting the intervention's ability to prevent future ED visits.

### 2.3 Setting

For the ED‐LEAD pragmatic clinical trial, the CPTI is delivered to PLWD who are discharged home from the ED and their identified care partners. Fourteen health systems with 79 EDs from across the United States participate in ED‐LEAD, with 40 sites assigned the CPTI. To deliver the CPTI, each ED site partners with an emergency medical services (EMS) agency to deliver the program. Many, but not all, EMS agencies participating in ED‐LEAD have an established community paramedicine program, leading to variation in experience.

### 2.4 CPTI Program Coordinator

The success of CPTI relies on a strong ED–EMS partnership; a skilled program coordinator is essential for facilitating referrals, maintaining intervention fidelity, and monitoring data. Program coordinators are typically research team members available during standard business hours. These coordinators are often health system employees, as they require access to the ED's electronic health record (EHR) to review patient information, assess eligibility, and complete the CPTI referral. If a program coordinator is not employed by the health system, then the site needs to ensure they can have access to the EHR. They also access CTI+ (Care Coordination Systems), the CPTI EHR designed specifically to support implementation of the Care Transitions Intervention (with modifications for CPTI). The client review tool within CTI+ is used by program coordinators to track fidelity to the intervention and follow up with coaches when needed. Program coordinators with strong communication skills, attention to detail, and experience in program monitoring are particularly well‐suited for this role.

### 2.5 Community Paramedicine and Community Paramedic Coach Selection

Community paramedicine, a community‐based health care model in which paramedics function outside their traditional emergency response and transport roles, has gained acceptance as an innovative, important, and effective new public and community health mission for paramedics [23, 24]. Researchers have demonstrated the feasibility, acceptability, and validity of community paramedicine‐delivered care activities ranging from administering vaccines to assessing homes for fall risk to enhancing care transitions, as well as improved outcomes for patients [25, 26]. Community paramedics are well‐suited to serve as CPTI coaches because they are highly trained, experienced in providing care in the home, respected by patients, and deeply embedded in their communities. To deliver the CPTI, paramedics must be currently licensed and employed by an EMS agency that is either internal to, or contracted by, the health system.

The success of CPTI largely depends on the effectiveness of community paramedics to coach the dyad. Paramedics with strong interpersonal skills, adaptability, and familiarity with navigating the healthcare system are particularly well‐suited for this role. To support EMS agencies in recruitment, our team developed a practical guide for paramedics considering becoming a coach. Developed by a community paramedic with extensive CPTI experience, the guide (see Supporting Information) underscores that coaching requires a fundamental role shift for paramedics. Rather than performing tasks for a patient, CPTI paramedic coaches must focus on empowering patients and care partners to take action themselves. By articulating this distinction, the guide assists paramedics in assessing their readiness for the role and promotes the self‐selection of candidates best aligned with the program's goals.

### 2.6 CPTI Training Curriculum

Paramedics complete a training program designed to equip them with the knowledge and skills necessary to deliver the CPTI. The curriculum incorporates independent and collaborative learning experiences designed for adult learners. An emphasis is placed on the coach's role as a “skill developer,” supporting dyads in building communication strategies and strengthening their confidence to manage future health care challenges. For the ED‐LEAD study, training is delivered to cohorts of participating paramedics, organized according to each site's implementation timeline. This cohort approach gives coaches the opportunity to learn from and support each other.

**FIGURE 2:**
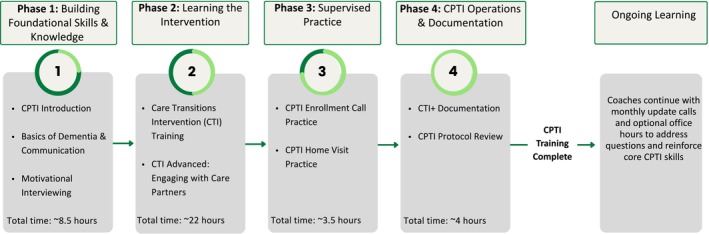
CPTI coach training curriculum overview. CPTI, Community Paramedic‐led Transitions Intervention.

The CPTI training curriculum is organized into four phases (Figure 2). The first phase introduces foundational content on motivational interviewing and dementia to establish core skills and knowledge. The second phase provides instruction on the Care Transitions Intervention coaching model central to the CPTI. The third phase includes virtual supervised practice of the enrollment call and the home visit, and the fourth phase contextualizes the intervention by emphasizing adaptations required for delivery by community paramedics to PLWD and their care partners. This phase also includes instruction on CPTI operations and documentation using the CTI+ EHR. Learning is reinforced through peer learning calls held after each phase, which provide opportunities to apply training content, address questions, and strengthen the integration of skills into practice. The curriculum combines asynchronous, self‐paced online modules with synchronous, virtual classroom sessions. The total estimated time commitment is 40 h over approximately 6 weeks. Table S1 describes the CPTI training curriculum in more detail, including the time commitment and objectives of each component. Coaches are required to successfully complete all CPTI training before becoming certified CPTI coaches.

Skill maintenance continues after the initial training through monthly sessions led by our experienced CPTI team, which includes emergency medicine physicians, prehospital medicine physicians, gerontological social workers, and a community paramedic with experience delivering the CPTI. These sessions foster a community of practice with peer‐to‐peer learning, troubleshoot challenging situations, and reinforce core CPTI skills. Coaches are required to participate in these monthly training sessions. In addition, peer‐to‐peer shadowing is encouraged as a strategy for giving and receiving feedback, with opportunities for new coaches to be observed by experienced colleagues to further refine their skills.

### 2.7 Socializing the CPTI Program to ED Staff

Because the CPTI is introduced to the dyad prior to discharge from the ED, ED providers, nurses, and other relevant staff must be engaged and supportive of the program, including understanding how to effectively introduce it and accurately convey its specifics to the dyad. To achieve these ends, it is crucial that the CPTI be socialized to ED staff before and regularly after launch until it is integrated into usual workflows. A variety of outreach materials have been found to be effective for this work, including CPTI signage in the ED, newsletters, staff meeting outreach, and emails from site leadership to ED staff encouraging participation in the program. Outreach efforts are recommended to begin roughly 4 weeks before the program's launch to give staff ample time to learn about the program and ask any clarifying questions. Additional outreach to staff after referrals begin further bolsters buy‐in and awareness of the CPTI. The outreach may include sharing data on the number of referrals, patient testimonials, and paramedic coach observations on the impact of CPTI. An example of a flyer used to help socialize the CPTI to ED staff is in the Supporting Information.

### 2.8 CPTI Workflow

**FIGURE 3:**
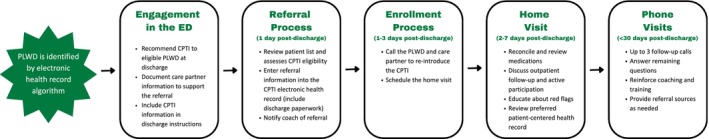
CPTI workflow for PLWD. CPTI, Community Paramedic‐led Transitions Intervention; ED, emergency department; PLWD, persons living with dementia.

The CPTI workflow begins in the ED (Figure 3), leveraging clinical decision support implemented into the EHR. Consistent with the other ED‐LEAD programs, patient identification for CPTI is based on a validated algorithm requiring one ICD‐10 diagnostic code for dementia documented in the EHR within the past 3 years [27, 28]. Depending on site capabilities, the workflow may vary. Table 1 details the core tasks involved in the CPTI workflow and common approaches taken by sites to accomplish these tasks. Below we describe the ideal CPTI workflow, tailored to the ED context and designed to maximize CPTI enrollment.

**TABLE 1:** CPTI ED and referral workflow for the ED‐LEAD project.

Task	Description	Options for EHR build
Identify PLWD	The EHR identifies any patient (age 66+) who meets the eligibility criteria of at least one ICD‐10 code diagnosis of dementia. The ICD‐10 code can be from an inpatient or outpatient encounter within the last 3 years of the ED visit. *Hospice patients should be excluded*.	Create a registry using Bynum code. Use Bynum code directly for each patient.
Select service area	Each site must define the CPTI service area based on its ability to deliver the intervention. Options include specific zip codes, county lines, etc.	If the service area can be limited by the EHR, the CPTI workflow could be limited to only patients eligible for the intervention. The program coordinator can manually determine whether a patient lives in the CPTI service area during the referral process.
Providers recommend CPTI to eligible PLWD at discharge	Patients are more likely to enroll in CPTI when it is recommended by their ED clinicians. For eligible patients, providers should receive an alert prompting them to discuss CPTI with the patient before discharge.	Interruptive disposition alert that displays in the disposition navigator for providers, ideally when they mark the patient for discharge. Non‐interruptive alert.
Document if a patient declines CPTI (ONLY IF REQUIRED)	The CPTI is a standard of care intervention and an extension of the patient's care in the ED. Some health systems might require documentation if the PLWD does not want to participate in CPTI. This likely *does not* apply if the EMS agency is housed within the health system.	Question added to disposition alert for providers. Question added to nursing documentation at discharge. Add a referral order to the disposition alert for providers. Include an option for “patient declined” if CPTI is not ordered.
Inform nurse of CPTI	The nurse discharging the patient will have to collect information on the patient's care partner and review CPTI information on the patient's discharge instructions. Before these steps are taken, it is recommended to inform the nurse that the patient qualifies for CPTI.	Add an order to the provider's disposition alert for an ED nurse communication. Recommended language: “Document care partner information for community paramedic referral AND highlight the CPTI referral in the discharge instructions.” Interruptive alert at discharge.
Collect care partner information	Before eligible patients are discharged home, information is needed about their care partner (e.g., name, phone number, interpreter needs) to make sure the CPTI program can reach them post‐discharge.	Care partner documentation included as a “current task” under the nurse communication order. Documentation occurs in the ED narrator.* Documentation included in nurse disposition navigator.* *Care partner documentation by any nurse is required before discharging the patient.
Share CPTI information with PLWD and care partner	Information about the CPTI should be included in the patient's discharge instructions to provide a reference for coaches when contacting the dyad (PLWD and care partner). The nurse should review the discharge instructions with the dyad before discharge, highlighting the CPTI information.	Information about the CPTI, including the EMS agency's contact information, should automatically populate to the patient's discharge instructions for all eligible patients. An optional CPTI flyer can also be attached to the patient's discharge instructions.
Post‐discharge: Refer eligible patients to CPTI	The program coordinator views the encounter‐level report and enters eligible referrals into CTI+ (the dedicated EHR for the CPTI), along with a copy of the patient's discharge instructions.	After an eligible patient is discharged from the ED, relevant information (e.g., names and contact information for the dyad) automatically populates to a report in the EHR. Manually populated encounter list stored on a secure drive.
Post‐discharge: Track CPTI referrals within the health system's EHR	Program coordinator documents referrals to CPTI and why patients were not referred (e.g., lives in a skilled nursing facility, outside CPTI service area, etc.).	CPTI referral screener to be completed within the EHR by the program coordinator for each eligible ED encounter.** Manually populated encounter list stored on a secure drive. **Information from the CPTI referral screener should populate back to the referral report (described above) for each eligible encounter.

Abbreviations: CPTI, Community Paramedic‐led Transitions Intervention; ED, emergency department; EHR, electronic health record; EMS, emergency medical services; PLWD, persons living with dementia.

#### 2.8.1 CPTI Workflow in the ED

Introducing the CPTI to the dyad before ED discharge is critical to support future enrollment. Given that paramedic coach‐led visits are rarely feasible in the time‐pressured ED, providers and nurses are encouraged to discuss the CPTI with patients and, when possible, their care partners. Providers are notified of a patient's eligibility through a disposition alert, driven by the EHR algorithm, during the discharge process. As part of this alert, the provider completes an order that alerts the nurse of the patient's CPTI eligibility and directs them to collect the care partner's name and phone number. The instructions to the nurse encourage them to ask the patient, “Do you have someone who helps you manage your health and other tasks at home, like a family member or friend that we can contact to support you after you are discharged?” Because this information is necessary for post‐discharge follow‐up by the paramedic coach, the discharge instructions cannot be printed until the care partner information is documented. We chose this approach, rather than using the primary contact, because the care partner for this illness or injury may not be the primary contact. After documenting the care partner information, the nurse can print the discharge instructions, to which a brief description of the CPTI and a program flyer featuring the CPTI coaches' first names, headshots, and contact information have already been added (see flyer example in the Supporting Information). The nurse can then review these materials with all eligible patients and their care partners.

#### 2.8.2 CPTI Referral Workflow

After an eligible patient is discharged from the ED, relevant information (e.g., names and contact information for the dyad) automatically populates to a report. This report is reviewed by the program coordinator daily, and they confirm that the PLWD is eligible for the CPTI. Our exclusion criteria include living outside of the CPTI service area, receiving hospice services, or being discharged to a skilled nursing facility. Sites can exclude ineligible patients earlier in the ED workflow (e.g., only flagging patients within the CPTI service area, excluding patients on hospice) if their systems can reliably do so. Typically, for eligible patients, the program coordinator completes the CPTI referral by documenting the CPTI referral outcome in the EHR, entering relevant information into CTI+ (including the care partner's contact information and relationship when known), uploading the patient's discharge instructions, and notifying the coach of the referral. Patients being discharged to an assisted living or a memory care facility are still eligible for the intervention. Due to facility rules and structure, additional targeted outreach to these facilities is encouraged to facilitate referrals/participation.

#### 2.8.3 CPTI Paramedic Coach Workflow

Once the paramedic coach receives the referral, they initiate enrollment by contacting the PLWD and their care partner to re‐introduce the CPTI and schedule a home visit, ideally within 2–7 days of ED discharge. At some sites, the program coordinator rather than the coach handles the enrollment call and home visit scheduling. Securing agreement from the dyad for a home visit can be challenging; therefore, coaches and program coordinators are trained in effective engagement strategies (e.g., linking the program to the patient's recent ED visit and referencing discharge instructions). Sites are also encouraged to ensure that their caller ID clearly identifies the health system or EMS agency, reducing the likelihood that dyads will mistake the enrollment call for a scam.

Once the members of the dyad are enrolled in the program, the coaching model involves helping them uncover their personal goals during the home visit and building motivation around the four pillars related to those goals over the 30‐day intervention period. During the home visit, the coach also conducts a structured medication review with the dyad, identifying and addressing discrepancies as appropriate. After the home visit, the coach provides up to three follow‐up phone calls to reinforce the four pillars of the intervention, address emerging concerns, and strengthen the dyad's ability to respond to new challenges. All intervention activities are completed within 30 days of ED discharge, with clinical documentation completed by the coaches in CTI+. Although CTI+ does not directly integrate with the health system's EHR, program coordinators can manually bridge the gap by exporting CPTI activity summaries from CTI+ and uploading them to the health system's EHR.

During the intervention, the coach approaches the dyad as a team and tries to engage the PLWD as much as possible depending on their current abilities. For example, if the patient has severe dementia, then the coaching would primarily be directed at the care partner; and if the patient has mild dementia, the coaching would primarily involve the PLWD. Successful coaching involves empowering the *dyad* to bridge the gap between clinical recommendations and daily life, ensuring they have the confidence and practical skills to manage their care at home. For instance, if a medication discrepancy is identified during the home visit, the coach does not correct the error themselves but rather guides the dyad through the process of contacting their provider or pharmacist to resolve it. During the first follow‐up call, the coach reviews the outcome of the medication discrepancy to validate the dyad's self‐management efforts and address any remaining barriers to adherence.

## 3 Intervention Monitoring

As shown in Table 2, the CPTI monitoring plan for ED‐LEAD is guided by the Reach, Effectiveness, Adoption, Implementation Fidelity, and Maintenance (RE‐AIM) framework [29, 30]. This framework provides a structured approach to assessing essential metrics required to ensure real‐world feasibility and long‐term sustainability in clinical practice. Weekly monitoring is conducted jointly by site leadership, study leadership, and the ED‐LEAD implementation core and includes review of program reach (e.g., proportion of eligible patients enrolled) and fidelity (e.g., proportion of medication reviews completed). Documentation in CTI+ facilitates fidelity assessment and implementation tracking. Program coordinators can monitor client‐level data and confirm completion of intervention steps from within CTI+. A daily updated dashboard provides site‐level performance information on key metrics, enabling teams to quickly identify potential issues. To ensure accurate and consistent data monitoring, the CPTI team trains site program coordinators to use these tools for fidelity review.

**TABLE 2:** Monitoring plan for the CPTI using the RE‐AIM framework.

Domain	Indicator/measure	Data source	Monitoring plan	Use of data
Reach	#, % of discharged patients referred #, % of eligible patients within the CPTI service area #, % of eligible patients enrolled #, % of eligible patients who complete the CPTI	Site EHR CTI+	Weekly by CPTI leads and site coordinators	Assess identification strategy and eligibility determination Identify barriers to enrollment and completion Troubleshoot solutions with sites when needed
Effectiveness	Reduction in ED return visits Intraindividual changes on the Patient Activation Assessment	Medicare claims data CTI+	Post‐implementation by study team Monthly by CPTI leads and site coordinators	Assess impact of CPTI on reducing site‐level ED return visits Identify barriers to patient activation
Adoption	#, % of paramedics who complete CPTI training #, % of paramedics who participate in update calls #, % of paramedics who serve as coaches for the full implementation period	Learning management system CPTI training tracker CPTI coach tracker	Ongoing by CPTI leads	Identify barriers to training, coach engagement, and retention Revise training curriculum as needed Troubleshoot with sites barriers to retention as needed
Implementation Fidelity	#, % of eligible patients with a home visit scheduled within 10 days of discharge #, % of eligible patients with a home visit completed within 15 days of discharge #, % of follow‐up phone calls completed within 30 days of discharge #, % of Patient Activation Assessment forms completed #, % of Medication Discrepancy Tools completed	CTI+	Weekly by CPTI leads and site coordinators	Identify barriers to timely completion and documentation of the CPTI Ensure patient activation is assessed and medication reviews are completed Troubleshoot with sites barriers to implementation fidelity, including coach competency
Maintenance and Adaptations	#, % of sites that continue CPTI after study completion Adaptations made to sustain the program	Site survey	Pre and post each implementation period by study team	Identify effective adaptations and maintenance strategies that can be shared across CPTI sites

Abbreviations: CPTI, Community Paramedic‐led Transitions Intervention; ED, emergency department; EHR, electronic health record; RE‐AIM, Reach, Effectiveness, Adoption, Implementation Fidelity, and Maintenance.

When concerns arise (e.g., low enrollment), the CPTI team works collaboratively with the site to diagnose contributing factors and co‐develop responsive strategies. These may include targeted retraining on specific intervention steps, adjustments to referral workflows to better identify eligible patients, enhanced socialization in the ED, or additional coach support. This structured, iterative support process is designed to restore and sustain high‐fidelity implementation while allowing sites to tailor delivery approaches to their local contexts.

## 4 Future Directions

While the CPTI offers a scalable framework for transitional care, its implementation within ED‐LEAD highlights additional avenues for further inquiry, particularly to focus services on patients who can benefit the greatest from the program and to determine how to most efficiently support PLWD. For instance, future research should investigate how the intervention's effectiveness varies across the spectrum of cognitive impairment and care partner involvement. Additionally, more evidence is needed to better understand how resource constraints and socioeconomic factors may impact the effectiveness of the intervention. Future studies should also examine the impact of CPTI on care partner outcomes and what specific mechanisms (e.g., faster outpatient follow‐up or medication reconciliation) contribute to success. Better understanding these clinical and contextual factors will help tailor the CPTI to the diverse needs of older adults and their care partners navigating the transition from the ED to home.

## 5 Conclusion

The CPTI offers a structured, feasible approach to supporting PLWD and their care partners during ED‐to‐home transitions. This paper described the CPTI conceptual model, workflow, paramedic coach training, and implementation monitoring framework, highlighting how these components work together to ensure fidelity and support dyad activation. While future research will continue to refine the model, the CPTI demonstrates the potential to improve post‐discharge outcomes and reduce avoidable ED visits. This framework can provide a model for health systems, provider groups, and EMS agencies interested in adopting this approach and implementing the CPTI.

## Author Contributions

Meghan Jenkins Morales contributed to the design, literature search, analysis, and manuscript preparation and editing. Meghan Jenkins Morales affirms all co‐authors listed contributed significantly to the work, and written consent was obtained by all contributors who are not authors and are named in the Acknowledgement section. Stephanie Ricketts contributed to the literature search, analysis, and manuscript editing. Corita R. Grudzen contributed to the concept development, design, analysis, and manuscript editing. Abraham A. Brody contributed to the concept development, design, analysis, and manuscript editing. Joshua Chodosh contributed to the concept development, design, analysis, and manuscript editing. Keith Goldfeld contributed to the concept development and manuscript editing. Manish N. Shah contributed to the concept development, design, literature search, analysis, and manuscript preparation and editing.

## Funding

Research reported in this publication was supported by the National Institute on Aging of the NIH under award number U19 AG078105‐01A1. This research was also funded in part through the National Cancer Institute P30 CA008748.

## Disclosure

Sponsors of this work had no role in the design, methods, subject recruitment, data collection, analysis, or preparation of this paper. This manuscript is the result of funding in whole or in part by the National Institutes of Health (NIH). It is subject to the NIH Public Access Policy. Through acceptance of this federal funding, NIH has been given a right to make this manuscript publicly available in PubMed Central upon the Official Date of Publication, as defined by NIH.

## Conflicts of Interest

The authors declare no conflicts of interest.

## Linked Article

This publication is part of the special collection titled *Emergency Departments LEading the transformation of Alzheimer’s and Dementia Care (ED‐LEAD)*. To view all articles under this special collection visit https://agsjournals.onlinelibrary.wiley.com/hub/journal/15325415/special‐collections.

## Supporting information


**Table S1:** CPTI training curriculum description and objectives.
