# Supplementary material for: Bridging the Gap Between the ED and Home: The Community Paramedic‐Led Transitions Intervention for Persons Living With Dementia
Source: J Am Geriatr Soc. 2026 Apr 15;74(7):1872–81. doi: 10.1111/jgs.70403 (PMC13403662; doi:10.1111/jgs.70403)
Supplement: Supplementary file 1 — Table S1: CPTI training curriculum description and objectives. [file JGS-74-1872-s001.pdf]

Is Becoming a CPTI Coach a Good Fit for Me?

A practical guide for paramedics considering this role

Written by: Gail Campbell, retired fire fighter/paramedic, PM2, community paramedic

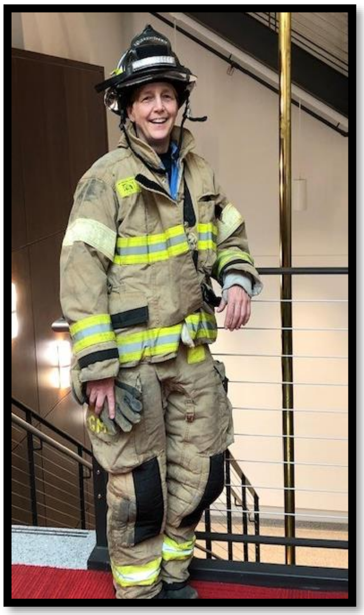

I came to community paramedicine after over 20 years as a full-time fire fighter/paramedic (FF/PM) for the Madison Fire Department (MFD). I was looking for new opportunities within the MFD and, luckily, the MFD was starting a community paramedic program. At first, I worked a hybrid schedule, one 24-hour shift as an FF/PM and two 10-hour days, as a community paramedic each week. Later, I became a full-time community paramedic because I found I really loved the new role. As a community paramedic, I have performed the Community Paramedic-led Transitions Intervention (CPTI), and I have worked as a coach with patients living with dementia and their care partners. Working with patients in my community in this new way felt like a chance for me to do my small part to not only improve people's experience with emergency medicine, but with healthcare in general.

The CPTI coach role may be an easy fit for paramedics currently working as community paramedics in their EMS agency; however, it may be something very new to your program. I believe that any paramedic considering the role of CPTI coach will learn a unique and valuable set of skills. I also think that this role comes more easily to paramedics with certain personality traits, and it is not for everyone. Below are some insights from my experience transitioning from a paramedic running EMS calls to a community paramedic supporting patients as a CPTI coach.

Coach Skills/Role:

- **This is** a significant role shift for paramedics who have spent years making fast decisions on emergencies, telling patients what they should do, following protocols and completing tasks quickly.

- **This is** a role in which you will "coach" patients with dementia and care partners (dyads) to help *build skills and empower* them to be able to manage their own medical affairs with more confidence. This is not a fast process. It may seem easier *for you* to just do it for them, but it is not better *for them*.

- **This is not** about paramedic coaches fixing the problem, but about helping dyads find the solution they need.

- **This is** working with older patients with dementia and their care partners in a *collaborative* way, allowing them to be the authority in their own lives and guide the

intervention toward their unique needs. Some of their needs may not be medical. This role uses a "start where the patient is at" approach.

- **This is** more of a "9-5" job in which you will visit patients and their care partners in their home once and call them up to 3 times in a 30-day period.

Coach Characteristics:

- **This is** for paramedics who enjoy talking with older adults, who consider themselves a "people person," and someone who is willing to forego what's easy in the name of supporting the dyad's long-term goals and growth.

- **This is** for paramedics who have patience, excellent verbal communication skills, flexibility, curiosity, willingness to learn new techniques, and a general love of humans with a soft spot in their hearts for older adults.

- **This can be** a great fit for paramedics with some experience (although it's not required!) as they bring an acute ability to assess medical issues and quickly see what's going on in someone's home. Not only do they have experience with medications/medical history/disease processes, but they also have a high level of comfort walking into any home and meeting people from all walks of life.

- **This is** for paramedics who are interested in learning new techniques to work with patients with dementia and their care partners, who are interested in helping patients to take more ownership of their healthcare and use the system more effectively and efficiently. This could be seen as "EMS prevention."

- **This is not** for paramedics who are feeling burned out from "over-utilizers." This work can actually be *more* challenging; thus, it may not be the break you are looking for.

- **This is not** for paramedics who thrive on being "action-oriented.". There is often not much action and a lot more talking. In fact, for paramedics who want to "get it done," this process could prove to be highly frustrating. (*Obviously, being a paramedic is action-filled, but, in this role, paramedics take that experience and use it to help patients take their own action).*

TABLE S1. CPTI training curriculum description and objectives.

Training Block	Delivery Method	Length	Attendees	Description and Objectives
Phase 1: Building Foundational Skills and Knowledge ~8.5 coach hours				
CPTI Training Call: Introduction	Synchronous Virtual Call	1 hour	Paramedics and Program Coordinators	The purpose of the CPTI introduction call is to "set the stage" and prepare coaches for the CPTI training process. At the end of the CPTI introduction block, coaches will be able to:<br>1. Recognize the need for improved care transitions from the ED to home among PLWD.<br>2. Differentiate between the CTI and CPTI model.<br>3. Describe the CPTI and how community paramedics can improve care transitions for PLWD.<br>4. Explain the ED-LEAD study and how the CPTI interfaces with the Emergency Care Redesign and Nurse-led Telephonic Care interventions.<br>5. Identify and explain the roles of key members of the CPTI team.<br>6. Summarize the CPTI training process and expectations.
Aliviado: Basics of Dementia	Online Training Course	2.5 hours	Paramedics	The purpose of the basics of dementia training is to establish foundational knowledge about dementia. At the end of the CPTI basics of dementia block, coaches will be able to:<br>1. Describe different types of dementia, their causes, and how dementia is different from normal aging.<br>2. Summarize the cognitive, psychological, and behavioral symptoms across the stages of dementia.<br>3. Describe respectful communication strategies that recognize and uphold the personhood of PLWD.

				4. Identify common acute changes among PLWD including delirium and common behavioral symptoms, and non-pharmaceutical intervention methods.<br>5. Recognize common challenges faced by care partners of PLWD and potential resources and strategies to address these challenges.
Dr. Nate Chin: Communication with PLWD	Online Training Course	1 hour	Paramedics	The purpose of the dementia communication training is to develop effective communication strategies for engaging PLWD and their care partners across different social, economic, and cultural backgrounds in the CPTI. At the end of the CPTI dementia communication training block, coaches will be able to:<br>1. Discuss how a diagnosis of dementia and dementia stage impact patient autonomy and decision-making capacity.<br>2. Describe the impact of stigma on the lived experience of PLWD and common biases related to working with PLWD.<br>3. Demonstrate effective communication when working with care partners and PLWD.
MI Companion	Online Training Course	3 hours	Paramedics	The purpose of the MI Companion training is for coaches to develop foundational motivational interviewing skills that will be expanded during the CTI training. After completing the MI online course, coaches will be able to:<br>1. Compare and contrast an MI approach to traditional directive communication styles.<br>2. Describe the benefits of using MI.<br>3. Explain the process to follow when building motivation.

				4. Generate open-ended questions to elicit “change talk.”<br>5. Practice reflective listening and other MI skills to empower the dyad.
CPTI Training Call: Communication	Synchronous Virtual Call	1 hour	Paramedics	Building from the dementia and MI training blocks, the purpose of the CPTI communication call is to apply MI strategies to working as a coach with the dyad. At the end of the CPTI Communication Training Call, coaches will be able to:<br>1. Appropriately modify communication strategies when working with PLWD at different stages of dementia and their care partner.<br>2. Explain the process of building motivation when working with PLWD and care partner.<br>3. Discuss additional considerations when using MI with the PLWD and their care partner.
Phase 2: Learning the Intervention ~22 hours				
CTI Pre-Training Course	Online Training Course	2 hours	Paramedics and Program Coordinators	The purpose of the CTI pre-training course is for coaches and Program Coordinators to begin to understand the CTI® model, which serves as the foundation for the CPTI. At the end of the CTI pre-training course, coaches will be able to:<br>1. Differentiate the Transitions Coach® role from other care team members.<br>2. Identify ways this model may differ from coaches' current way of interacting with the people they are serving.<br>3. State what the Four Pillars® are.<br>4. Discuss the value of Skill Transfer®.<br>5. Use new strategies to enhance client communication.

				6. State why goal setting is important to a patient-centered approach.
CTI Learning Platform Orientation	Synchronous Virtual Call	1 hour	Paramedics	The purpose of the CTI learning platform orientation is for coaches to become familiar with the Adobe Connect platform used during the CTI Virtual Classroom Training. This training is required by Care Coordination Systems before the CTI Virtual Classroom Training.
CTI Training	Synchronous Virtual Calls	16 hours	Paramedics	The purpose of the CTI training is for coaches to learn the CTI model by applying and practicing coaching skills and techniques. At the end of the CTI virtual classroom training, coaches will be able to:<br>1. Demonstrate each component of the Care Transitions Intervention®.<br>2. Demonstrate understanding of Skill Transfer®.<br>3. Be confident in the completion of a Medication Discrepancy Tool.<br>6. Identify what skills to coach the patient in during the intervention.<br>7. Demonstrate program fidelity through role-play scenarios and participation.<br>8. Identify network resources as well as local and national support.<br>9. Identify ways to stay up to date in coach skills, post-training support, and resources.
CTI Advanced: Engaging with Care Partners When Conducting CTI	Online Training Course	3 hours	Paramedics	The purpose of this advanced training is to help a certified Transitions Coach learn to effectively engage familial care partners when conducting the CTI. At the end of this training, coaches will be able to:

				1. Describe the different types and roles of care partners as well as recognize common challenges care partners face.<br>2. Apply specific strategies to involve care partners in the 30-day CTI intervention that promotes skill development within 4 key areas of health (medication management, red flags, medical care follow-up appointments, and maintaining a personal health record).<br>3. Apply communication techniques to engage both the care partner and the PLWD, including in-person and over the phone.<br>4. Identify strategies to help care partners engage in self-care and respite to prevent burnout.<br>5. Understand how to leverage the skills and contributions of familial care partners while promoting person-centered care to achieve more efficient health care during transitions.
Phase 3: Supervised Practice ~3.5 coach hours				
CPTI Training Call: Enrollment Call Practice	Synchronous Virtual Call	1.5 hours	Paramedics and Program Coordinators*	The purpose of the CPTI enrollment call practice is to prepare CPTI coaches to independently conduct the enrollment call with the dyad. At the end of the CPTI enrollment call practice session, coaches will be able to:<br>1. Describe the essential elements of the enrollment call.<br>2. Apply appropriate strategies for building rapport/trust and engaging the dyad in the CPTI over the phone.

				3. Demonstrate how to build enthusiasm for the CPTI with the dyad over the phone and successfully schedule a home visit.
CPTI Training Call: Home Visit Practice	Synchronous Virtual Call	2 hours	Paramedics	The purpose of the CPTI home visit practice is to provide coaches with additional time to practice the skills and techniques necessary for a CPTI home visit. At the end of the CPTI home visit practice session, coaches will be able to:<br>1. Apply CTI coaching techniques to a mock home visit.<br>2. Demonstrate competency in Skill Transfer®, goal identification, and coaching across the Four Pillars®.<br>3. Appraise skills demonstrated by peers and offer constructive feedback.
Phase 4: CPTI Operations and Documentation ~4 coach hours				
CTI+ Documentation Training	Synchronous Virtual Call	3 hours	Paramedics and Program Coordinators (first hour)	CTI+ is the EHR platform used to document client information, encounter notes, and program outcomes for the CPTI program. The purpose of the CTI+ Documentation Training is to introduce coaches and Program Coordinators to the platform through a guided demonstration and an interactive, hands-on assignment. By the end of the CTI+ Documentation Training, coaches will be able to:<br>1. Access and navigate the CTI+ platform using their Okta account credentials.<br>2. Understand key documentation tools, fields, and requirements within CTI+.<br>3. Enter client information and encounter notes accurately and in alignment with program standards.<br>4. Complete and interpret multiple Patient Activation Assessments® and Medication Discrepancy Tools® to monitor and compare clients' progress over time.<br>5. Identify workflow or documentation challenges and communicate them for implementation support.

				6. Access Customer Success resources to troubleshoot issues or request assistance.
CTI+ for Program Coordinators and Supervisors	Synchronous Virtual Call	1.5 hours	Program Coordinators and other Site Supervisors	The purpose of this training is to equip CPTI program coordinators and supervisors with the knowledge and skills needed to effectively oversee client documentation and workflows within the Care Coordination Systems documentation platforms. By the end of this session, supervisors will be able to:<br>1. Understand the Client Health Record framework and identify how their agency and role fit within it.<br>2. Differentiate between account types (Coach, Supervisor, and Hub Manager) and describe the access levels and functions available to each.<br>3. Perform key supervisory tasks and workflows in the CHR, including creating and assigning client records, updating client status, conducting quality improvement checks, and closing out records.<br>4. Locate appropriate resources for support, including workflow-specific guidance and CTI+ platform assistance.
CPTI Training Wrap-Up Call: Protocols to Practice	Synchronous Virtual Call	1 hour	Paramedics and Program Coordinators	The purpose of the CPTI training wrap-up call is to review key protocols, reinforce core concepts from previous sessions, and address any remaining questions before implementation. Coaches and Program Coordinators will confirm their understanding of CPTI procedures, timelines, and expectations to ensure readiness for program launch. By the end of this training session, both groups will be able to:<br>1. Assess coach readiness to implement the CPTI intervention in alignment with program protocols and quality standards.<br>2. Review and demonstrate understanding of key CPTI protocols, including client enrollment timelines, follow-up expectations, and intervention completion deadlines.

				3. Discuss remaining implementation questions and share insights or challenges with instructors and peers to support successful program launch.<br>4. Describe available resources to support coaches while implementing the CPTI.
Ongoing Learning Post Training				
CPTI Update Calls	Synchronous Virtual Calls	1 hour	Paramedics and Program Coordinators*	The purpose of the CPTI update calls is to provide ongoing training, implementation support, and peer learning opportunities for coaches and program coordinators. These calls offer programmatic updates, educational opportunities, skill-building, and a forum to discuss lessons from the field, ensuring consistent, high-quality delivery of the CPTI intervention across sites. By the end of each CPTI update call, participants will be able to:<br>1. Stay informed about key program updates, reminders, and announcements related to CPTI.<br>2. Strengthen knowledge and skills related to CPTI implementation through targeted training and guest presentations.<br>3. Collaborate with peers to discuss real-world challenges, share lessons learned, and identify best practices for improving participant outcomes.
CPTI Office Hours	Synchronous Virtual Calls	1 hour	Paramedics* and Program Coordinators*	The purpose of CPTI office hours is to provide an open, supportive space for coaches and program coordinators to discuss challenging cases, clarify program requirements, and receive guidance to strengthen CPTI implementation and data quality. By the end of CPTI office hours, participants will be able to:<br>1. Discuss and problem-solve complex or challenging CPTI cases with input from peers and facilitators.<br>2. Clarify questions related to CPTI protocols, data entry, and program implementation.

				3. Identify strategies and resources to improve program delivery and participant outcomes.
CPTI Data Monitoring	Synchronous Virtual Call	1 hour	Program Coordinators	The purpose of the CPTI data monitoring call is to strengthen program coordinators' skills in accurately entering, reviewing, and interpreting CPTI data to ensure data quality, timely reporting, and effective program oversight. By the end of the training, program coordinators will be able to:<br>1. Describe their role in CPTI data monitoring, including accurate referral entry and consistent data review practices.<br>2. Identify and verify key CPTI data elements for completeness and accuracy.<br>3. Apply CPTI data deadlines to ensure timely and compliant data entry and reporting.<br>4. Interpret ED-LEAD site-level real-time data reports to track program progress and identify data quality issues.<br>5. Differentiate between the CHR Caseload Grid and CTI+ Client Review Report and explain how each tool supports data monitoring.

Note: * Indicates that the training block is optional for this group.
CPTI, Community Paramedic–Led Transitions Intervention; CTI, Care Transitions Intervention; ED, emergency department; ED-LEAD, Emergency Departments LEading Transformation of Alzheimer's and Dementia Care; EHR, electronic health record; EMS, emergency medical services; MI, motivational interviewing; PLWD, persons living with dementia.

Training Compliance and Documentation

All CPTI coaches must complete all CPTI training before being allowed to implement the intervention. Participation in additional post-training update calls is required, and attendance at Office Hours is strongly encouraged. The Care Transitions Intervention Program providers will monitor and provide quality assurance during the Care Transitions Intervention virtual training course. In addition, Care Transitions Intervention offers monthly community learning calls (separate from CPTI update calls) as ongoing support for all Care Transitions Intervention-trained coaches.

Attendance will be taken for all synchronous training sessions. Community paramedics are strongly encouraged to attend trainings as scheduled at the start of the implementation period. If a paramedic is unable to make a scheduled synchronous training session, arrangements will be made for them to view a recording and complete a short assessment or schedule a make-up training.

Competence in and completion of online, asynchronous training modules will be tracked via completion of skills assessments. The research team will maintain records of coach training completion across all sites.

Insert Health System logo here

CPTI

Community Paramedic-led Transitions Intervention

Insert EMS partner logo here

WHAT IS IT?

- CPTI is a 30 day **coaching** program led by insert EMS Agency **community paramedics** including 1 home visit and up to 3 follow-up phone calls.
- CPTI is **free** for patients and care partners!

WHY DOES IT MATTER?

- This is a chance to expand our **care** beyond the ED!
- It supports families navigating **dementia** and helps reduce unnecessary ED returns or hospitalizations.
- Your **participation** is key to the program's success.

HOW DOES THIS HELP PATIENTS?

- This program supports patients in their **homes**.
- Patients and their care partners will gain skills related to **medication** management, navigating the **healthcare system**, and responding appropriately to their conditions.

WHAT DO WE NEED FROM YOU?

- Talk to patients about CPTI and **encourage** participation.
- Collect **care partner** information to support the referral process and **connect** eligible patients to this program.
- Highlight the **CPTI referral** in the discharge paperwork.

QUESTIONS OR FEEDBACK?

- Please reach out to insert primary contact with questions or suggestions.
- Go-live is insert date!

Insert Health System logo here

CPTI

Insert EMS partner logo here

Community Paramedic-led Transitions Intervention

WHAT IS IT?

- CPTI is a 30 day **coaching** program for **insert ED site** patients led by your **local paramedics at insert EMS agency.**
- CPTI is **free** for patients and loved ones!

WHY SHOULD I PARTICIPATE?

- Research suggests that the CPTI helps **prevent** unnecessary ED return visits.
- CPTI coaches can work with your **schedule**.
- CPTI is designed to keep you in **control** of your health.

WHAT SHOULD I EXPECT?

- **1 call** from a paramedic shortly after your discharge from the ED to schedule **1 home visit**.
- Personalized coaching for **30 days** to discuss your goals.
- Up to **3 follow-up calls** to help you manage your health needs.

WHAT WILL WE DISCUSS?

- Strategies to **manage medications**.
- **Outpatient follow-up** with primary or specialty care.
- Maintaining and communicating a **personal health record**.
- **Responding** appropriately to your health conditions.

WHERE CAN I FIND MORE INFORMATION?

MEET OUR PARAMEDICS

Insert Coach Photo

Insert coach name & phone number

Insert Coach Photo

Insert coach name & phone number

Insert Coach Photo

Insert coach name & phone number
